# Morphological and Hemodynamic Discriminators for Rupture Status in Posterior Communicating Artery Aneurysms

**DOI:** 10.1371/journal.pone.0149906

**Published:** 2016-02-24

**Authors:** Nan Lv, Chi Wang, Christof Karmonik, Yibin Fang, Jinyu Xu, Ying Yu, Wei Cao, Jianmin Liu, Qinghai Huang

**Affiliations:** 1 Department of Neurosurgery, Changhai Hospital, Second Military Medical University, Shanghai, China; 2 Cerebrovascular Center, Department of Neurosurgery, Houston Methodist, Houston, Texas, United States of America; University of Washington, UNITED STATES

## Abstract

**Background and Purpose:**

The conflicting findings of previous morphological and hemodynamic studies on intracranial aneurysm rupture may be caused by the relatively small sample sizes and the variation in location of the patient-specific aneurysm models. We aimed to determine the discriminators for aneurysm rupture status by focusing on only posterior communicating artery (PCoA) aneurysms.

**Materials and Methods:**

In 129 PCoA aneurysms (85 ruptured, 44 unruptured), clinical, morphological and hemodynamic characteristics were compared between the ruptured and unruptured cases. Multivariate logistic regression analysis was performed to determine the discriminators for rupture status of PCoA aneurysms.

**Results:**

While univariate analyses showed that the size of aneurysm dome, aspect ratio (AR), size ratio (SR), dome-to-neck ratio (DN), inflow angle (IA), normalized wall shear stress (NWSS) and percentage of low wall shear stress area (LSA) were significantly associated with PCoA aneurysm rupture status. With multivariate analyses, significance was only retained for higher IA (OR = 1.539, p < 0.001) and LSA (OR = 1.393, p = 0.041).

**Conclusions:**

Hemodynamics and morphology were related to rupture status of intracranial aneurysms. Higher IA and LSA were identified as discriminators for rupture status of PCoA aneurysms.

## Introduction

With wider availability of advanced imaging techniques, incidental findings of unruptured intracranial aneurysms are increasing. Once an unruptured aneurysm is diagnosed, the risk of rupture has to be balanced against the risk of treatment, which highlights the importance of accurate assessment of rupture risk [1, 2]. Although a wide range of clinical, morphological and hemodynamic parameters are believed to contribute significantly in determining the rupture risk of intracranial aneurysms, studies on predictors of aneurysm rupture showed conflicting findings [3–5]. Confounding factors may include differences in nature history and perianeurysmal environment of aneurysms in different locations [6]. Focusing on a single location may therefore avoid bias in the results. Recent studies on the natural history of intracranial aneurysms showed that posterior communicating artery (PCoA) aneurysms accounted for approximately 15%–25% of all intracranial aneurysms and were more prone to rupture [7]. Few studies has focused on only PCoA aneurysms by comprehensively considering clinical, morphological and hemodynamic features [8]. Here, we compared clinical, morphological and hemodynamic characteristics of ruptured and unruptured PCoA aneurysms to determine possible discriminators of aneurysm rupture status.

## Materials and Methods

The Institution Review Board of Changhai Hospital, Second Military Medical University approved this retrospective study and the requirement for informed consent was waived. The patients’ information was anonymized and de-identified prior to analysis. In addition, we have not conducted research outside of our country of residence.

### Patients and Clinical Characteristics

From January 2012 to December 2013, 129 consecutive patients with PCoA aneurysm were diagnosed in our hospital by three-dimensional rotational angiography (3DRA). To correctly determine the rupture status of the PCoA aneurysms, patients with multiple aneurysms were excluded. Among the 129 PCoA aneurysms, 85 were ruptured and 44 were unruptured according to the history of subarachnoid hemorrhage (SAH). Data on the following clinical characteristics were collected: age, sex, medical history (hypertension, diabetes mellitus, use of antithrombotic agents before SAH), smoking history (currently smoking or not), history of familial aneurysmal SAH, presence of homolateral oculomotor nerve palsy, fetal type PCoA or not. Hypertension was defined as taking antihypertensive agents, a systolic blood pressure of ≥ 140 mm Hg, or a diastolic blood pressure of ≥ 90 mm Hg before the onset of SAH. Diabetes mellitus was defined as taking antidiabetic agents, treatment with insulin injection, a fasting plasma glucose level of ≥ 126 mg/dl, a random plasma glucose level of > 200 mg/dl, or a hemoglobin A1c level of ≥ 6.5%. A fetal-type PCoA was defined as a PCoA that has the same or larger caliber as the P2 segment of the posterior cerebral artery and is associated with an atrophic P1 segment.

### Imaging Acquisition and Morphological Parameters

The 3DRA examinations were performed with an Artis zee Biplane angiographic system (VC14, Siemens AG, Erlangen, Germany). A 5-second digital subtraction angiography (DSA) acquisition protocol was adopted, and 18 mL of contrast agent was injected through the internal carotid artery at a rate of 3 mL/s. During the 5-second acquisition after a delay of 1 second, a 200° rotation of the C-arm was performed to obtain 133 frames. All of the acquired 5-second DSA data were transferred to a *syngo* X Workplace (VB15, Siemens AG) workstation for reconstruction of the 3D internal carotid arterial tree and exported in a stereolithography (STL) format for computational fluid dynamics (CFD) simulations.

**Fig 1:**
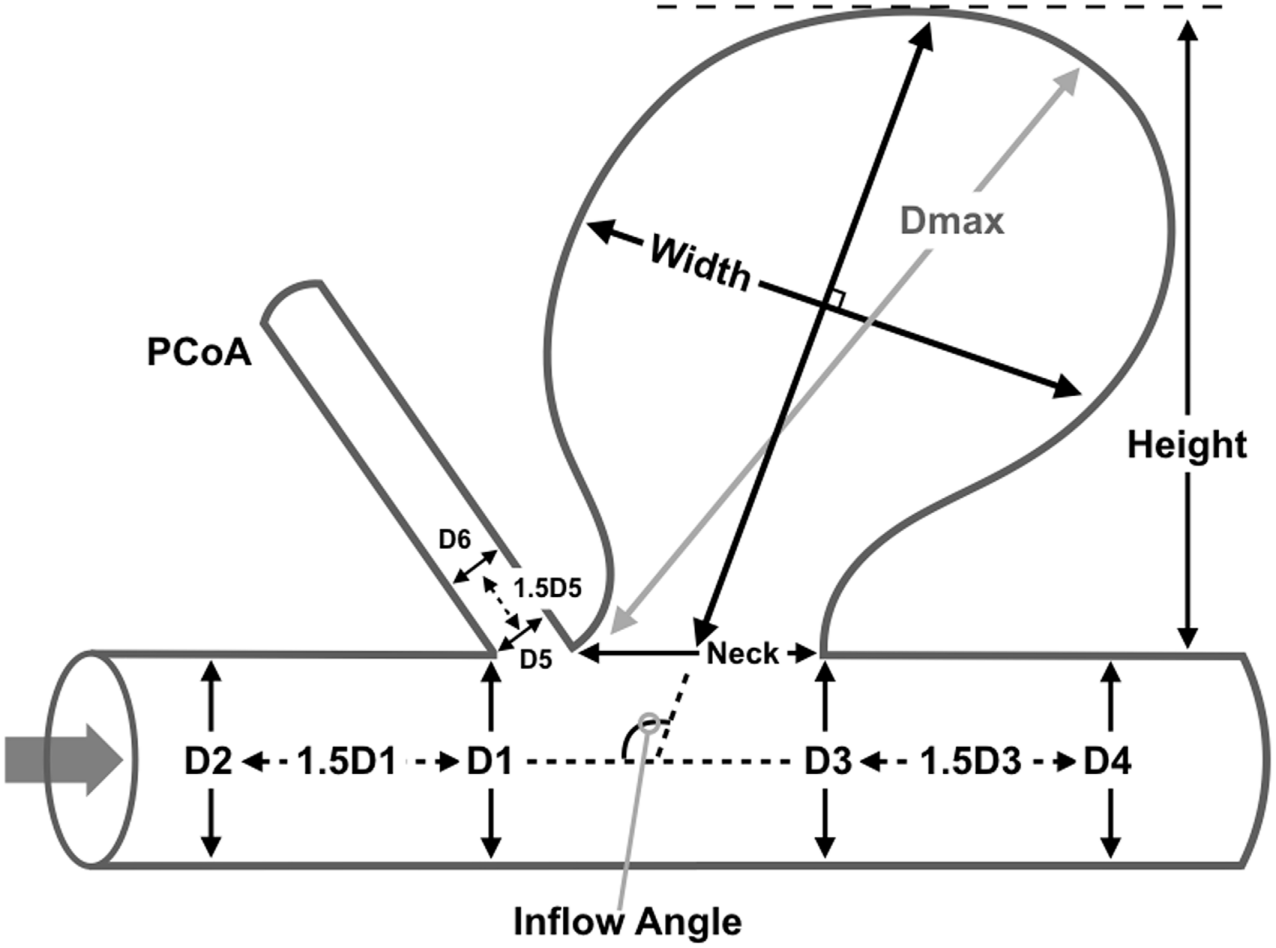
Definitions of Morphological Parameters. Size of aneurysm dome = Dmax; aspect ratio (AR) = Height/Neck; size ratio (SR) = 6Dmax/(D1+D2+D3+D4+D5+D6); dome-to-neck ratio (DN) = Dmax/Neck; bottleneck factor (BN) = Width/Neck.

Morphological parameters of the PCoA aneurysms were defined as previous studies (Fig 1) and calculated using GEOMAGIC STUDIO 9.0 software (Geomagic, Morrisville, North Carolina) and Matlab (The Math Works, Inc., Natick, MA, USA)[4, 9]. Size of aneurysm dome was the maximum diameter of the aneurysm dome. Dome height was the longest dimension from the neck to the dome tip and dome width was measured perpendicular to the dome height. Aspect ratio (AR) was calculated by dividing dome height by neck width. Size ratio (SR) was computed by dividing size by the average diameter of parent arteries and dome-to-neck ratio (DN) by dividing size by neck width. Bottleneck factor (BN) was defined as the ratio of dome width to neck width. Inflow angle (IA) was the angle between the axis of proximal parent artery and the aneurysm’s main axis from the center of the neck to the tip of the dome.

### Computational Fluid Dynamics (CFD) and Hemodynamic Parameters

The 3D STL models were segmented and smoothed by GEOMAGIC STUDIO 9.0 software (Geomagic, Morrisville, North Carolina) and imported into ICEM CFD 11.0(ANSYS, Lebanon, New Hampshire) to create volume grids for CFD simulation. The number of total elements of each model was approximately between 1,800,000 and 2,200,000. The vessel wall was divided into 3 parts: aneurysm, parent artery and other vessels, as previously described [9].

CFD simulations were performed with CFX 11.0 (ANSYS) by solving the governing Navier-Stokes equations assuming rigid walls, no-slip boundary conditions, laminar and incompressible blood flow (density ρ = 1050kg/m^3^ and viscosity μ = 0.00345 Pa·s). A pulsatile velocity profile was imposed at the inlet derived from transcranial Doppler (TCD) and the outlet was defined as an open boundary with zero static pressure. The cardiac cycle (duration 0.8 seconds) was discretized by a time-step of 0.001 seconds. For each model, three continuous cardiac cycles were simulated to ensure the numerical stability of the simulation and results were presented from the last cycle. The results were post-processed and visualized with CFX 11.0 (ANSYS).

Three hemodynamic parameters were calculated in this study: normalized wall shear stress (WSS), percentage of low WSS area (LSA) and oscillatory shear index (OSI). The time-averaged WSS was averaged over the dome area (the entire luminal surface of the aneurysm sac) and then normalized by the average parent vessel WSS in the same patient to obtain normalized WSS (NWSS), which aims to allow comparison among different patients [5, 10]. LSA, defined as the areas of the aneurysm wall exposed to a WSS below 10% of the mean parent vessel WSS, was then normalized by the dome area [5, 10]. OSI, a non-dimensional parameter that measures the directional change of WSS during the cardiac cycle[11]:

$$WSS\;=\;\frac{1}{T}\int_{0}^{T}\left|wss_{i}\right|dt$$ (1)

$$OSI=\frac{1}{2}\left\{1-\frac{\left|\int_{0}^{T}wss_{i}dt\right|}{\int_{0}^{T}|wss_{i}|dt}\;\right\}$$ (2)

where wss_i_ is the instantaneous WSS vector and T is the duration of the cycle. The OSI was averaged over the dome area.

### Statistical Analysis

Statistical analysis was performed with Microsoft Excel 2003 and SAS 9.1(SAS Institute Inc, Cary, NC, USA). Variables were expressed as median (interquartile range), or number of patients (%) as appropriate. The chi-square test was performed for cross-tabulation. For the measured data, Mann-Whitney U-test was used. The parameters found to be significant in univariate analysis were further analyzed using multivariate logistic regression (backward elimination) to identify those that retained significance when accounting for all relevant parameters. The observed range of each parameter was scaled to a range from 0 to 10, so that a unit increase in the parameter corresponded to 10% of its observed range. We then calculated the AUC-ROC on the predicted probability of rupture status from the regression models. P<0.05 (two sided) was the criterion for statistical significance.

## Results

### Univariate Analysis between Ruptured and Unruptured Groups

**Fig 2:**
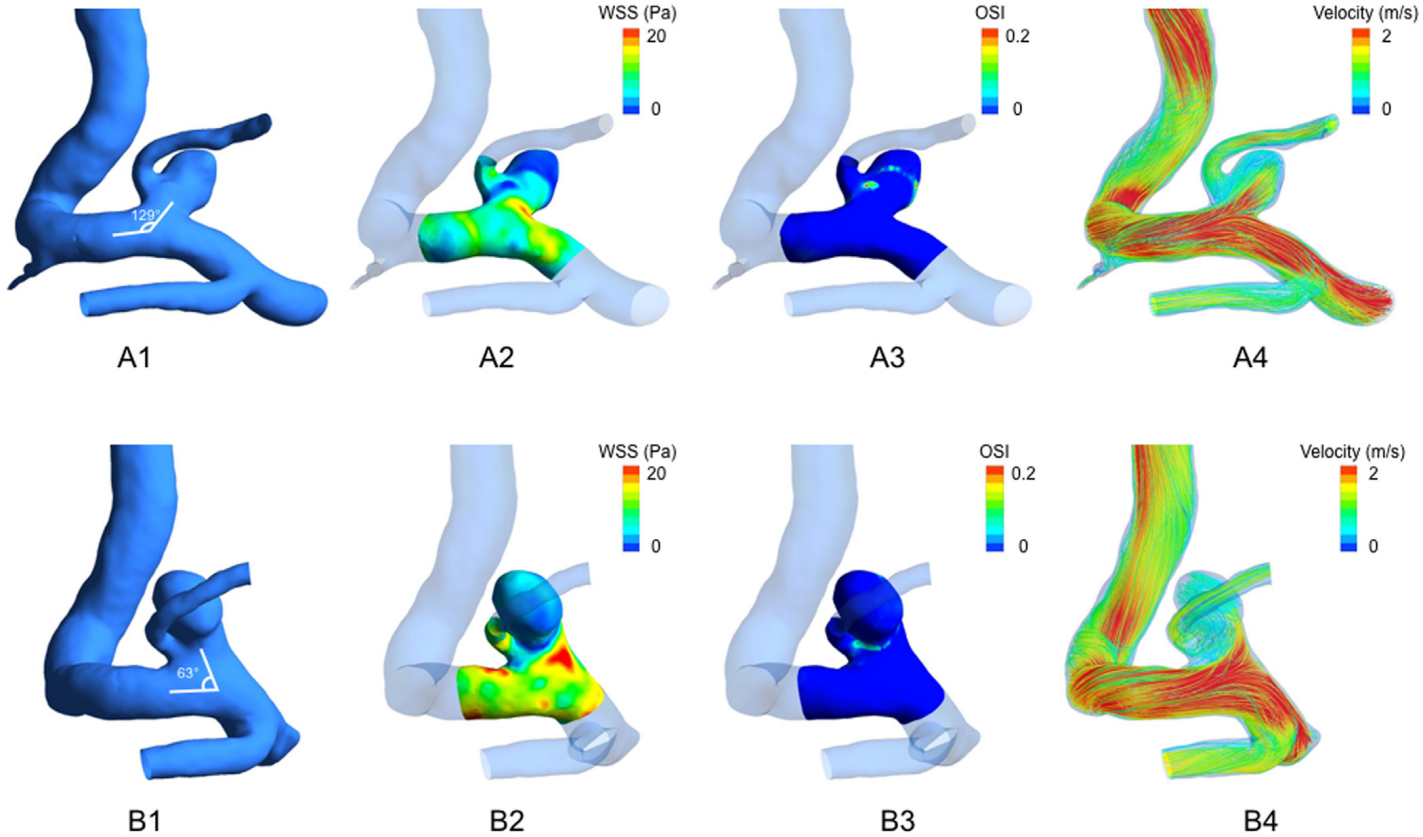
Hemodynamic patterns of two representative posterior communicating artery (PCoA) aneurysms. A: ruptured PCoA aneurysm with a higher inflow angle (IA, 129°); B: unruptured PCoA with a lower IA (63°). From left to right: (1) Three-dimensional models and measurement of IA; (2) distribution of wall shear stress (WSS); (3) distribution of oscillatory shear index (OSI); (4) flow pattern at the systolic peak.

The clinical, morphological and hemodynamic characteristics of PCoA aneurysms are shown in Table 1. The patients’ ages ranged from 42 to 82, with a mean age of 59.9 years (29 or 22.5% were males). No clinical variables included in this study showed significant difference related to rupture status of the aneurysm (p>0.05). Morphological parameters AR (p = 0.002), SR (P = 0.001), DN (p<0.001), IA (p<0.001) and hemodynamic parameters NWSS (p = 0.002), LSA (p<0.001) were found significantly different between the ruptured and unruptured aneurysm groups. Other morphological and hemodynamic parameters, including BN (p = 0.080) and OSI (p = 0.183) revealed no significance. Hemodynamic patterns of two representative cases are shown in Fig 2.

**Table 1:** Clinical, morphological and hemodynamic characteristics of PCoA aneurysms.

	Ruptured	Unruptured	
Variables	N = 85	N = 44	P Value
**Baseline Characteristics**			
Age	58 (41, 64)	62 (55, 67)	0.118
Male	18 (21.2)	11 (25.0)	0.660
Hypertension	38 (44.7)	22 (50.0)	0.568
Diabetes mellitus	5 (5.9)	7 (15.9)	0.063
Antithrombotic agent	6 (7.1)	6 (13.6)	0.368
Current smoking	6 (7.1)	2 (4.5)	0.860
Familial SAH	11 (12.9)	5 (11.4)	0.797
Oculomotor nerve palsy	14 (16.5)	3 (6.8)	0.124
Fetal type PCoA	29 (34.1)	19 (43.2)	0.313
**Morphological Characteristics**			
Size, mm	4.864 (3.960, 6.563)	4.251 (2.622, 5.398)	0.002
Aspect ratio (AR)	1.180 (0.892, 1.516)	0.862 (0.689, 1.128)	<0.001
Size ratio (SR)	1.857 (1.416, 2.462)	1.461 (0.907, 1.885)	0.001
Dome-to-neck Ratio (DN)	1.263 (0.991, 1.735)	0.990 (0.829, 1.206)	<0.001
Bottle-neck Ratio (BN)	1.185 (1.036, 1.416)	1.095 (0.896, 1.346)	0.080
Inflow angle (IA)	119.7 (104.3, 132.3)	98.7 (89.3, 118.1)	<0.001
**Hemodynamic Characteristics**			
Normalized WSS (NWSS)	0.562 (0.415, 0.827)	0.791 (0.580, 1.014)	0.002
Oscillatory shear index (OSI)	0.016 (0.010, 0.034)	0.015 (0.005, 0.025)	0.183
Percentage of low WSS area (LSA)	0.029 (0.001, 0.086)	0.004 (0.000, 0.032)	<0.001

Variables were expressed as median (interquartile range), or number of patients (%).

SAH, subarachnoid hemorrhage; PCoA, posterior communicating artery aneurysm; WSS, wall shear stress.

### Multivariate Logistic Regression on the Significant Parameters

Multivariate logistic regression was performed to identify the discriminators of PCoA aneurysm rupture status using a backward elimination process. All the significant parameters that were significant in univariate analysis were included. Statistical significance of IA and LSA were retained in this regression model. For a unit increase of the IA, the odds of rupture status increased by 1.539, for a unit increase of LSA, the odds of rupture status increased by 1.393 (Table 2). The ROC value was 0.764 (95%CI 0.673–0.854, p<0.001).

**Table 2:** Multivariate logistic regression analysis of parameters associated with rupture status of PCoA aneurysms.

Variables	Odds Ratio	95%CI	p value
Inflow angle	1.539	1.239–1.911	<0.001
Percentage of low WSS area (LSA)	1.393	1.013–1.915	0.041

PCoA, Posterior communicating artery aneurysm; WSS, wall shear stress.

## Discussion

The increasing numbers of incidental findings of unruptured intracranial aneurysms necessitates a more accurate approach of assessing rupture risk. Previous studies have highlighted the potential for predicting the rupture risk of intracranial aneurysms by morphological and hemodynamic assessment [8, 10, 12]. However, the results of current studies are conflicting. Large variations in the application of the CFD technique and the large number of parameters derived from it together with a heterogeneity of the investigated cases were not differentiated by clinical risk factors nor by the location of the included aneurysms, particularly in earlier studies. Focusing on a certain kind of aneurysm in patients with similar clinical data might therefore help reducing the effect of confounding influences for the application of CFD [13–15]. PCoA aneurysms are one of the most common aneurysms and more likely to rupture [1, 2, 7]. For the above stated reasons, we only considered PCoA aneurysms in our study.

Clinical characteristics were thought to be important factors affecting rupture status of aneurysms [8, 16]. But previous studies have not obtained consistent results. Patient age for instance has been reported to be either correlated or anti-correlated with aneurysm rupture risk [13, 14, 16]. The inconsistent findings might be caused by the differences of patient selection and the multiple-location design. Therefore, in this study, in addition to restricting aneurysm location, also clinical characteristics were balanced between the ruptured and unruptured groups, with no significant difference between them to further help eliminating bias in patient selection for the morphological and hemodynamic results.

An increasing number of morphological parameters have been introduced to discriminating rupture status of aneurysms [4, 17]. Size was believed to be a significant parameter to predict rupture risk of unruptured aneurysms. Forget et al [18] reviewed the sizes of ruptured aneurysms and found that 85.6% of these aneurysms were less than 10 mm. The prevalence of small ruptured PCoA aneurysms was particularly high with 87.5% of aneurysms measuring less than 10 mm in diameter and 40% measuring less than 5 mm. In addition to size, we also screened other morphological parameters that had been reported to be correlated with rupture risk, including AR, SR, DN, BN and IA. Although some qualitative parameters, like irregular shape [14] and direction of dome [13], were believed to be discriminators for PCoA aneurysm rupture status, they were not included in this study, as their criteria vary according to observers. In the univariate analysis, size, AR, SR, DN and IA were significantly higher in the ruptured group, while in the multivariate logistic regression, only IA was retained as a morphological discriminator in the regression model for rupture status. Being different with parameters like AR that focused on the geometry of the aneurysm dome itself, IA descripted the spatial relationship of the aneurysm dome to its parent vessels, which affect the hemodynamic pattern inside the aneurysm dome to some extent [6, 19–21]. Baharoglu et al [22] revealed that increasing IA in bifurcation aneurysms reduced brisk flow in the dome and limited secondary flow to the sides of the aneurysm dome near its neck, which resulted in much lower WSS. This is consisted with our results that higher IA and lower WSS status were observed in ruptured PCoA aneurysms.

Controversy still exists as to the role of WSS on aneurysm formation and rupture. Cebral et al [23] argued that WSS of ruptured aneurysms were higher than that of unruptured aneurysms in a study of 210 aneurysms, while Xiang et al [5] demonstrated lower WSS was independent risk factor for aneurysm rupture on the basis of morphological-hemodynamic analysis of 119 intracranial aneurysms from multiple locations. The hemodynamic patterns may be influenced by the great morphological variations of different anatomic location aneurysms [20]. It might be more reasonable to perform hemodynamic analysis focusing on a single location of aneurysms. Miura et al [15] and Zhang et al [14] revealed a correlation between low WSS and aneurysm-rupture status in middle cerebral artery aneurysms and PCoA aneurysms respectively. Also in a previous hemodynamic study focusing on PCoA aneurysms, we demonstrated low WSS status in aneurysms that were prone to rupture [24]. These location-specific studies have highlighted the important role that low WSS plays in aneurysm rupture. Extraordinarily low WSS may up-regulate endothelial surface adhesion molecules, trigger inflammatory pathways and cause degradation of the aneurysm wall that could ultimately lead to rupture [25].

In addition to WSS, OSI is another important hemodynamic in aneurysm development [5]. Higher OSI may damage the luminal surface and promote the processes of leukocyte adhesion, smooth muscle proliferation, and vasoconstriction. This extracellular milieu, through its action onto the cytoskeletal filaments via its anchorage of the cell membrane to the substrate, the nucleus or even neighboring cells, influences cell biology [26]. In our results, the value of OSI in the ruptured group was higher than the unruptured group, but with no significant difference. According to a recent systematic review and meta-analysis of hemodynamic factors, the distribution of OSI showed that ruptured aneurysms did not have significantly different pooled OSI compared with unruptured aneurysms, while high OSI was significantly correlated with aneurysm formation [27]. This may suggest a more important role of high OSI during aneurysm formation than during aneurysm rupture.

## Limitations

The study has several limitations. Firstly, although patient-specific models were used, we did not use patient-specific boundary conditions because of the retrospective study. Second, morphology of the PCoA aneurysms may change after aneurysm rupture, which could affect the accuracy of hemodynamic analysis. Lastly, the logistic regression model was developed only for PCoA aneurysms, which cannot be applied to aneurysms with other anatomic locations. The stability and efficiency of the model will be further tested in the future research.
